# Evaluation of a longitudinal subspecialty clinic for internal medicine residents

**DOI:** 10.1080/10872981.2021.1955429

**Published:** 2021-07-29

**Authors:** Martin V. Consunji, R. Jeffrey Kohlwes, Jennifer M. Babik

**Affiliations:** a Resident Physician, Department of Medicine, Keck School of Medicine of the University of Southern California; b Chief of the Division of General Internal Medicine, Department of Medicine, San Francisco Veterans Affairs Medical Center, University of California–San Francisco; Co-Director, PRIME Residency Program; c Division of Infectious Diseases, Department of Medicine, University of California–San Francisco; Associate Director for Subspecialty Education, Internal Medicine Residency Program; Assistant Director for Curriculum, Infectious Diseases Fellowship Program

**Keywords:** Educational continuity, internal medicine residency, subspecialty education, subspecialty choice, longitudinal mentorship, ambulatory education

## Abstract

**BACKGROUND:**

The traditional model for subspecialty education in internal medicine (IM) residencies is a short inpatient consult rotation, which often lacks outpatient exposure and continuity with faculty. Our IM residency program developed a longitudinal subspecialty clinic (LSC) experience, which pairs categorical IM residents with a faculty preceptor in their subspecialty of interest. Residents work in their preceptor’s clinic for one half-day per week during ambulatory blocks throughout the PGY2 year.

**OBJECTIVE:**

To evaluate the LSC program’s educational impact and determine best practices for successful implementation. METHODS: From May to July 2019, we surveyed residents and preceptors who participated in an LSC between 2014 and 2019, gathering quantitative and qualitative data on their experiences

**RESULTS:**

Survey response rates were 66.4% (N=93/140) for residents, 57.7% (N=15/26) for preceptors. Most residents and preceptors were very or extremely satisfied with their LSC experience (83.3% and 71.4%, respectively). Most residents and preceptors reported that the LSC experience was very or extremely effective in enabling residents to explore their subspecialty of interest (76.0%, 86.7%), form a mentoring relationship with their preceptor (71.3%, 80.0%), obtain a letter of recommendation (76.1%, 64.3%), prepare for fellowship (76.3%, 66.7%), gain exposure to outpatient subspecialty practice (90.0%, 73.3%), and gain medical knowledge (84.6%, 80.0%).

**CONCLUSIONS:**

Our data showed that LSCs are effective in facilitating longitudinal subspecialty career exploration, mentorship, and education for residents. Opportunities for improvement include developing a more structured curriculum, addressing scheduling issues, and adding the option to extend the experience to the PGY3 year.

## Introduction

Exposure to subspecialty medicine remains a core component of internal medicine (IM) residency training, irrespective of career path [1–4]. The traditional model of subspecialty education in IM residency, especially for categorical residents, is a two- to four-week rotation on an inpatient primary or consult service [5–7]. However, these experiences may not offer the full spectrum of subspecialty medicine, as most subspecialty careers include some component of outpatient practice [8,9]. In addition, these experiences may not offer continuity with faculty given the complexity of resident and faculty schedules and the block organization of traditional rotations[10]. There has been increasing interest in including ambulatory subspecialty experiences in IM residency training [1,5,7,11], but the ideal structure of such experiences is unknown[12].

In order to develop an innovative ambulatory subspecialty experience for our residents, we utilized the conceptual framework of educational continuity[10]. There is a wealth of literature in undergraduate medical education identifying the importance of continuity of supervision in clinical education[10]. In longitudinal experiences, learners are better able to build trusting relationships with clinical supervisors, and supervisors are able to provide more individualized education as they can better gauge the progression of a learner’s knowledge and skills over time [13–15].

To increase ambulatory exposure to subspecialty medicine in a manner that would foster the principles of educational continuity, we created the Longitudinal Subspecialty Clinic (LSC) program for second-year categorical residents (R2s/PGY2s) in our residency program. In the LSC program, each PGY2 is paired with a faculty preceptor in his/her subspecialty of interest, and the resident sees patients in the preceptor’s clinic throughout the ambulatory months of the PGY2 year. The objectives of the LSCs are to provide residents with: (a) improved exposure to ambulatory subspecialty diseases, (b) increased opportunities for career exploration in the ambulatory setting, and (c) opportunities to develop a strong mentoring relationship with their subspecialist preceptor. We here report our evaluation of the LSC program between 2014 and 2019 from the perspective of both residents and faculty preceptors. We will describe the program’s effectiveness in achieving its objectives as well as best practices for successful implementation.

## Methods

### Setting, participants, and program description

The LSC program was originally implemented in 2010 as part of the clinical research track (PRIME program) [16] within the larger categorical IM residency and then expanded in 2018 to include all categorical PGY2s. The residency program has ~44 categorical residents and ~18 primary care residents in each class, for a total of ~62 residents per class. The LSCs occur at one of the three affiliated teaching hospitals at the University of California–San Francisco (UCSF): UCSF Medical Center, Zuckerberg San Francisco General Hospital, and the San Francisco Veterans Affairs Medical Center. Categorical PGY2s have the option to participate in an LSC, and 92.7% of them participated between 2014 and 2019. Residents are paired with a faculty preceptor based on their stated subspecialty of interest. The LSC is built into residents’ schedules after their primary care clinic sessions (3 half-days per week) have been assigned. Each resident rotates in their preceptor’s subspecialty clinic for 1 half-day per week during ambulatory block months (6 months total during the PGY2 year), except when they are on vacation or inpatient elective rotations (~4-6 weeks per year). As a result, residents each have a total of ~16-18 LSC sessions. Over the 5-year study period (2014–2019), 140 residents were paired with one of 26 faculty preceptors (several faculty preceptors worked with multiple residents per year). There is no uniform curriculum across the LSCs; rather, the experience is determined by each resident-preceptor pair. Of note, UCSF primary care IM residents have a similar longitudinal experience in a ‘second clinic’ outside of their primary care clinic – some of which occur in subspecialty clinics – but these were excluded from our current study.

### Survey study design

From May to July 2019, we surveyed UCSF categorical IM residents (current and alumni) as well as UCSF subspecialty faculty preceptors who participated in an LSC between 2014 and 2019. Two online surveys (Supplemental Material), one for residents and one for preceptors, were developed with Qualtrics Survey Software (Provo, UT; Seattle, WA) using Artino’s survey design process [17] and then were distributed electronically. Survey validity was assessed using cognitive interviewing and pilot testing. The resident survey comprised 39 items and was sent to past and present resident participants of the LSCs over the 5-year study period, including 39 current PGY2s (class of 2020), 28 current PGY3s (class of 2019), and 73 alumni who had graduated in 2016–2018 (total of 140 residents). Of note, we chose not to include alumni who participated in an LSC between 2010 and 2014 (classes of 2012–2015) due to the increased risk of recall bias. The preceptor survey comprised 38 items and was sent to 26 LSC preceptors. In addition to items on demographics, both surveys consisted of 30 parallel items aimed at assessing career exploration, mentorship, education, and logistical hurdles in the LSC program[18], enabling direct comparison of resident and preceptor responses. Item types included multiple choice, five-point Likert scale, open-ended, and integer slider scale. Participation for both groups was voluntary and anonymous, and no incentives were provided for either group. The study was certified as exempt by the UCSF Institutional Review Board on 23 April 2019 (Reference #: 247952).

### Data analysis

Quantitative survey data was statistically analyzed using Prism Software (version 8.3.0; GraphPad; San Diego, CA). Comparisons between groups were made using the unpaired t-test or two-sided Fisher’s exact test, as appropriate. A *P* value of less than .05 was used to determine statistical significance. Qualitative survey data was first independently analyzed by two members of our research team (M.C., J.B.) using inductive content analysis to identify themes[19]. Codes were created separately and reconciled through iterative discussion.

## Results

### Survey respondents

**Figure 1.**
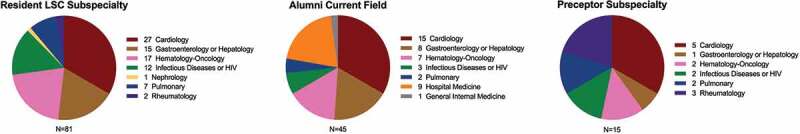
The distribution of specialty area is shown for residents (subspecialty of their LSC), alumni (current field of practice), and preceptors (subspecialty)

Of the 140 residents (67 current residents and 73 alumni) invited to complete our survey, 93 responded (66.4% total response rate), including 48/67 current residents (71.6% response rate) and 45/73 alumni (61.6% response rate). Of the 26 preceptors invited to complete our survey, 15 responded (57.7%). The demographic information for these resident and preceptor respondents is shown in Table 1 and Figure 1.

**Table 1.** Respondent Characteristics

Characteristic	N (%)
**Residents (N = 93)**	
**Current residents**	48 (51.6)
PGY2	28 (58.3)
PGY3	20 (41.7)
**Alumni**	45 (48.4)
Fellow	35 (77.8)
Attending	10 (22.2)
**Preceptors (N = 15)**	
Faculty rank	
Clinical instructor	1 (6.7)
Assistant professor	2 (13.3)
Associate professor	8 (53.3)
Professor	4 (26.7)
Years of experience as LSC preceptor	4.47 ± 4.75^a^
Number of residents precepted^b^	2.50 ± 1.12^a^

Data are shown as the number (%) of respondents unless otherwise indicated.

Abbreviations: PGY2, second-year resident; PGY3, third-year resident; LSC, Longitudinal Subspecialty Clinic; SD, standard deviation.

^a^Data shown as mean ± SD.

^b^Data shown is for the academic year at the time of our study (2018–2019).

### Overall evaluation

**Figure 2.**
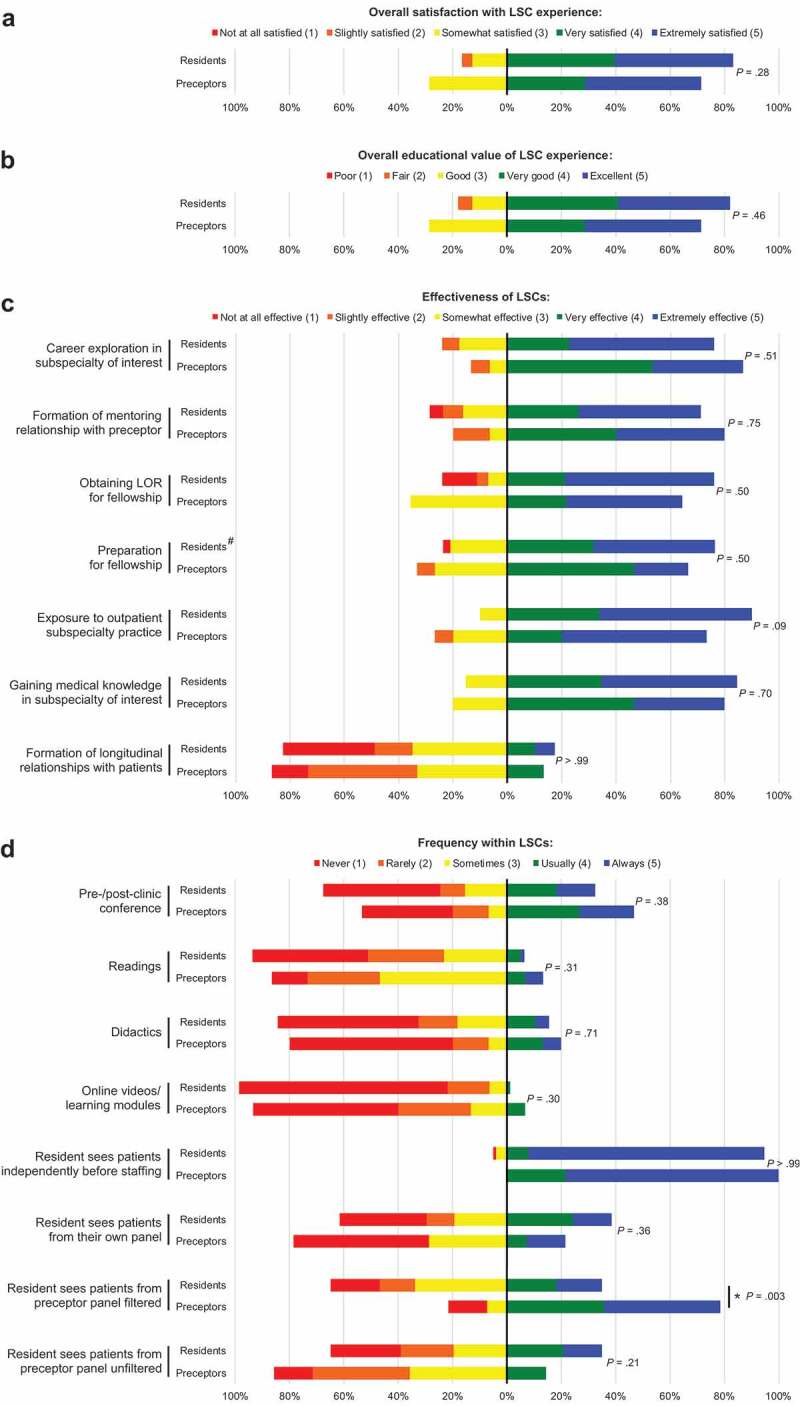
The distributions of resident and preceptor responses to Likert items for **(a)** overall satisfaction, **(b)** overall educational value, **(c)** effectiveness, and **(d)** curriculum and workflow. Bars to the right of the vertical baseline (0% axis) show the percentage of residents or preceptors who answered category 4 (green) or 5 (blue); bars to the left show the percentage who answered category 1 (red), 2 (orange), or 3 (yellow). The *P* values shown are for comparing the percentage of residents who answered category 4 or 5 with the corresponding percentage of preceptors. The pound sign (#) indicates an item posed only to alumni

83.3% (N = 65/78) of residents and 71.4% (N = 10/14) of preceptors reported being very or extremely satisfied with their LSC experience (Figure 2A). Additionally, there was no statistically significant difference in overall satisfaction when residents were stratified based on their LSC subspecialty (data not shown), although numbers were limited for some subspecialties (N = 1 for nephrology, N = 2 for rheumatology). 82.1% (N = 64/78) of residents and 71.4% (N = 10/14) of preceptors rated the overall educational value of the LSC experience as very good or excellent (Figure 2B). There were no statistically significant differences between residents and preceptors in these responses or in the subsections below unless otherwise noted.

Resident and preceptor responses to open-ended items addressed some of the strengths (career exploration, outpatient exposure, longitudinal mentorship) and areas for improvement (structured curriculum, scheduling, continuity patient panel) of the LSC program. Themes and representative quotes are summarized in Table 2.

**TABLE 2.** Results from Content Analysis of Responses to Open-Ended Items

**Theme**	**Representative Quotes**
***Strengths***	
Career exploration in subspecialty of interest	*Resident*: “I was interested in pursuing ID as a career and thought that a subspecialty clinic would be good exposure to the field.” *Preceptor*: “Cultivating interest through increased exposure and expertise.”
Exposure to outpatient subspecialty practice	*Resident*: “I was able to explore what outpatient cardiology looks like and gain perspective on the full spectrum of cardiac care.” *Resident*: “It was one of the most useful experiences during residency and was actually very helpful in preparing me for fellowship. It allowed a much smoother transition when I had my own busy clinic in fellowship to have had this unique experience.” *Preceptor*: “Rheumatology has a very large outpatient component (majority of rheumatology occurs in the clinic) and the LSC gives a resident a real glimpse of the patients we care for.”
Facilitation of subspecialty career choice (confirmation of interest, ruling out a subspecialty)	*Resident*: “It confirmed my clinical interest in pulmonary medicine.” *Resident*: “Realized that GI fellowship was not the best fit for me and decided on cardiology instead.” *Resident*: “I really appreciated the flexibility with changing my LSC in my second year after I switched my choice of specialties.”
Access to longitudinal subspecialty mentorship (faculty, fellows, research opportunities)	*Resident*: “It provided the opportunity for me to develop a great mentorship relationship with my clinic preceptor which I found helpful in applying into the subspecialty.” *Resident*: “Helped me meet and form relationships with fellows.” *Resident*: “Helped me access mentorship and research projects in cardiology.” *Preceptor*: “Felt like I could really invest in [residents] as learners, teach a lot of topics over time, show them an ‘approach’ to thinking about our diseases that plays out over months, rather than just teaching digestible topic-based items. And most of all, chance to form an academic bond and relationship was great, and talk to them over the course of months about our field, careers, life, etc., was excellent.”
Obtaining LOR for fellowship	*Resident*: “The only good way to get LORs from clinical subspecialists at [our institution]. This is essential to the fellowship application process, as our elective rotations in inpatient subspecialties do not give you the same exposure and would be very stressful if needed as a source of LORs.” *Preceptor*: “This year I will be writing three letters of recommendation for fellowship.”
Value in residents seeing patients from their preceptor’s panel unfiltered	*Resident*: “It worked great just having me see people as they came in. I was expected to keep up with the pace of the fellows and share the work in the clinic. I really enjoyed being treated as a member of the team, not being specifically scheduled for certain ‘resident-level’ patients.” *Preceptor*: “There is also a value in seeing the unfiltered bell curve of all patients presenting to clinic.”
More well-rounded training for residents planning generalist careers	*Resident*: “I’m interested in learning more about oncology, and though I’ll be a generalist I think many of the concepts are applicable.” *Resident*: “My LSC helped me to understand my love for ID within general medicine. Throughout the experience, I was constantly thinking of how I would apply the knowledge I obtained in clinic to my primary care practice. It helped me to see that I was already envisioning myself as a primary care physician with a focused interest in ID.”
***Areas for improvement***	
Desire for a structured, resident-level curriculum across the LSCs	*Resident*: “[The ideal LSC curriculum includes] online videos/modules for core topics (to allow consistency regardless of clinic).” *Resident*: “The sessions I attended were often targeted to the fellows and slightly above my knowledge level.” *Preceptor*: “[An area for improvement is] clear goals/objectives provided in advance from the medicine residency so that the experience is more uniform across different clinics.”
Preference for residents to see patients either from their own panel or from their preceptor’s panel filtered	*Resident*: “It would be nice to know further in advance who I would be seeing to be able to prepare more efficiently, and having patients on my schedule would be very convenient.” *Resident*: “I think seeing patients off the attending’s schedule allows for flexibility. If attendings are encouraged to set aside high yield patients, that could be helpful in ensuring a positive experience.” *Preceptor*: “[The ideal LSC workflow is for residents to] have their own small panel that they follow longitudinally.” *Preceptor*: “We try to filter patients for highest educational value to residents.”
Scheduling issues	*Resident*: “It’s challenging to skip in and out of the clinic as much as we do already because of vacation.” *Resident*: “I already have a decreased number of sessions due to vacations, 3-day weekends (my LSC falls on Monday).” *Preceptor*: “Keeping up with the schedules can be challenging – I’ve had days where I’ve been surprised to have a resident.” *Preceptor*: “[An area for improvement is] a more consistent resident presence. It’s too unpredictable to want to invest significantly.”

Abbreviations: GI, gastroenterology; ID, infectious diseases; LOR, letter of recommendation; LSC, Longitudinal Subspecialty Clinic.

### Subspecialty career exploration and choice

The majority of residents and preceptors reported that the LSC experience was very or extremely effective in enabling residents to explore their subspecialty of interest (76.0% [N = 60/79] and 86.7% [N = 13/15], respectively) (Figure 2C). When asked how their LSC influenced their career choice, the majority of residents explained that their experience either confirmed their subspecialty interest or helped them rule out a subspecialty (Table 2). 81.0% (N = 34/42) of current residents said they are planning to practice in the same subspecialty as their LSC, while 89.7% (N = 35/39) of alumni actually joined the same subspecialty. Although numbers were limited, when residents were stratified based on this career choice (practicing in the same subspecialty as their LSC or not), there were no statistically significant differences in responses on learner satisfaction or program effectiveness (data not shown). The only exception was that residents who changed subspecialty choice after their LSC rated the experience as less helpful in obtaining a letter of recommendation for their fellowship application (25.0% [N = 1/4] vs. 79.1% [N = 53/67]) (*P* = .04).

### Longitudinal subspecialty mentorship

The majority of residents and preceptors reported that the LSC experience was very or extremely effective in enabling residents to form a mentoring relationship with their preceptor (71.3% [N = 57/80], 80.0% [N = 12/15]) and obtain a letter of recommendation for fellowship (76.1% [N = 54/71], 64.3% [N = 9/14]) (Figure 2C). 80.0% (N = 12/15) of preceptors felt like they got to know their current resident(s) well enough to write them a letter of recommendation. Most residents (especially alumni) felt that the unique strength of the LSC program was the longitudinal mentorship, as it facilitated securing a strong letter of recommendation, getting involved with research projects, and creating a successful fellowship application (Table 2).

### Subspecialty education

The majority of residents and preceptors reported that the LSC experience was very or extremely effective in enabling residents to prepare for fellowship (76.3% [N = 29/38], 66.7% [N = 10/15]), gain exposure to outpatient subspecialty practice (90.0% [N = 72/80], 73.3% [N = 11/15]), and gain medical knowledge in their subspecialty of interest (84.6% [N = 66/78], 80.0% [N = 12/15]) (Figure 2C). Table 2 includes quotes from alumni that elaborate on how the LSC program helped prepare them for fellowship.

### Curriculum

Only a minority of residents and preceptors reported various curricular components as usually or always occurring within their LSCs (pre-/post-clinic conference: 32.5% [N = 25/77], 46.7% [N = 7/15]; readings: 6.4% [N = 5/78], 13.3% [N = 2/15]; didactics: 15.6% [N = 12/77], 20.0% [N = 3/15]; online videos/learning modules: 1.3% [N = 1/78], 6.7% [N = 1/15]) (Figure 2D). Many residents and preceptors expressed a desire for a more formal and uniform LSC curriculum (Table 2).

### Logistics

A majority of residents and all preceptors reported that residents usually or always see patients independently before staffing with their preceptor (94.9% [N = 74/78], 100.0% [N = 14/14]) (Figure 2D). A minority of residents and preceptors reported that residents usually or always see patients from their own resident panel (38.5% [N = 30/78], 21.4% [N = 3/14]). While only 35.1% (N = 27/77) of residents reported that they usually or always see patients from their preceptor’s panel selected based on perceived educational value, 78.6% (N = 11/14) of preceptors reported that this usually or always occurs in their LSC (*P* = .003). A minority of residents and preceptors reported that residents usually or always see patients from their preceptor’s panel selected solely based on workflow (35.1% [N = 27/77], 14.3% [N = 2/14]).

Residents reported that they saw a mean of 3.26 patients (SD = 0.95) each half-day LSC session, while preceptors reported a mean of 2.71 patients (SD = 0.61) for their residents (*P* = .04). Residents reported the ideal number of patients per session to be 3.23 (SD = 0.81); preceptors reported it as 3.14 (SD = 0.66) (*P* = .69).

A minority of residents and preceptors reported that the LSC experience was very or extremely effective in enabling residents to form longitudinal relationships with patients (17.5% [N = 14/80], 13.3% [N = 2/15]) (Figure 2C). The perception of how many patients were seen longitudinally over the year differed between residents and preceptors: residents reported seeing a mean of 4.55 patients (SD = 3.20) more than once over the course of the year, while preceptors reported that their residents saw a mean of 6.69 patients (SD = 5.50) more than once (*P* = .06).

**Figure 3.**
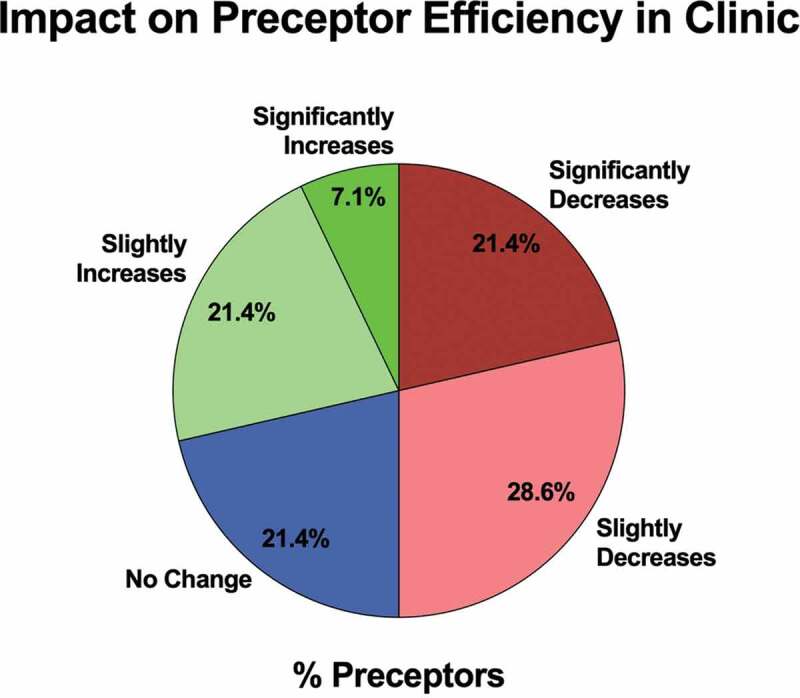
The impact of having an LSC resident on preceptor efficiency in clinic, with data shown as the distribution of preceptor responses

When asked about the impact of having a longitudinal resident on their efficiency in clinic, 21.4% (N = 3/14) of preceptors reported a significant decrease in efficiency, 28.6% (N = 4/14) a slight decrease, 21.4% (N = 3/14) no change, 21.4% (N = 3/14) a slight increase, and 7.1% (N = 1/14) a significant increase (Figure 3). Table 2 includes additional faculty perspectives on the benefits and challenges of participating as an LSC preceptor.

When asked how frequently they felt like the LSC experience was interrupted due to competing scheduling demands placed on residents, 10.3% (N = 8/78) of residents and 21.4% (N = 3/14) of preceptors reported that their experience was usually or always interrupted (*P* = .36). Table 2 includes quotes from residents and preceptors that highlight some of these scheduling issues (vacations, long holiday weekends, unpredictable resident LSC attendance).

97.1% (N = 67/69) of residents and 81.8% (N = 9/11) of preceptors preferred that a one-year LSC experience occur during the PGY2 year (versus the PGY3 year). 92.1% (N = 70/76) of residents and 76.9% (N = 10/13) of preceptors wanted the LSC experience to be extended to two years (during the PGY2 and PGY3 years). Moreover, 75.0% (N = 57/76) of residents would want to have the option to switch to a different LSC for the PGY3 year to experience another subspecialty.

## Discussion

Our study demonstrated that the LSCs were effective in facilitating subspecialty career exploration, mentorship, and education for residents. Residents and preceptors felt that potential areas for improvement include developing a more structured curriculum across the LSCs, addressing scheduling issues, and adding the option to extend the LSC experience to the PGY3 year.

Although 7% of USA IM residency programs in the mid-1990s offered ambulatory subspecialty continuity experiences similar to the LSCs[20], there remains a paucity of literature describing such experiences and their effectiveness. Our study builds upon prior studies of similar experiences for primary care IM [21] and pediatric [22] residents, as well as of an online endocrinology continuity clinic simulation for IM residents[5]. Our results are consistent with previous findings that mentorship has an important influence on career choice [23] and that educational continuity can better facilitate clinical mentorship [10,13–15]. The longitudinal design of the LSC allows residents to develop a more meaningful mentoring relationship with a subspecialist, who can serve as an invaluable resource for career decision-making and the fellowship application process – including writing a strong letter of recommendation. Moreover, as an experience based in the outpatient setting, the LSC gives residents a more complete picture of a subspecialty and allows them to make better-informed career choices. The LSC program is also worthwhile for residents planning careers in primary care or hospital medicine, as some residents felt that participating in an LSC made them better generalists.

Similarly to previous studies [21,22], we found that the LSC was an effective way to deliver subspecialty education to residents, which is consistent with the educational continuity framework[10]. By providing residents with the opportunity to participate longitudinally in a community of subspecialty practice, the LSC promotes learning and the beginning of the formation of a professional identity within that subspecialty[24]. However, in contrast to other studies [5,22], our results showed that the LSC did not effectively enable residents to establish patient care continuity. It is important to note that different medical subspecialties have varying degrees of continuity of care and that resident scheduling constraints likely contributed to this lack of continuity.

We found that there was some tension between the inherent flexibility of the LSCs and the desire for more structure across the program. Because the curriculum and workflow are determined by each resident-preceptor pair – and largely based on pre-existing logistics within a given subspecialty clinic – faculty are able to become LSC preceptors and integrate a resident into their clinic with relative ease. However, this variability creates a barrier to setting clear expectations for both residents and preceptors across the LSCs. Possible interventions to clarify expectations and provide more structured curriculum include standardizing orientation to the clinics, creating and tracking case/topic logs, and developing formal didactics in the form of brief lectures, videos, or online modules. The heterogeneity of LSCs also naturally leads to some scheduling issues, which may be mitigated by improving communication around schedules between the residents and preceptors.

The desire of most of the respondents to change the LSC curriculum into a two-year experience is a testament to the program’s popularity. It also presents an opportunity to address some program challenges. Two-year participation in an LSC would facilitate the creation of resident continuity patient panels and motivate the development of subspecialty-focused learning materials. Furthermore, the potential option for residents to switch to a different LSC for the PGY3 year would further facilitate either career exploration/choice for residents who are still deciding between multiple subspecialties or ambulatory exposure to another subspecialty.

Our study has several limitations. There was no concurrent control group because 93% of residents participated in an LSC. Similarly, we did not compare to a pre-intervention group of residents, given the LSC was first implemented in 2010 and using a historical control group could have led to the introduction of non-contemporaneous control bias. However, our qualitative data – particularly from alumni, having recently completed the LSC program and transitioned to fellowship/subspecialty practice – allowed us to gain a rich understanding of the impact of the LSCs, thus bolstering our survey conclusions. Another potential limitation is the generalizability of our findings. Our residents have six months of ambulatory time during the PGY2 year, which facilitates scheduling of the LSC. Other residency programs with less curricular time devoted to the outpatient setting may have more difficulty providing their residents with a similar experience. Yet, implementation of a program that facilitates *longitudinal* exposure to a subspecialty mentor in clinic, regardless of the specific number of ambulatory weeks/months, can likely achieve similar outcomes to our LSC program.

Finally, with many resident subspecialty clinics adopting telehealth (video and/or telephone visits) during the ongoing COVID-19 pandemic[25], our LSC model is still working with virtual precepting.

The LSC program was effective in achieving its objectives of providing categorical IM residents with longitudinal subspecialty career exploration, mentorship, and education in the ambulatory setting. With changing Accreditation Council for Graduate Medical Education (ACGME) program requirements calling for a subset of resident experiences to be individualized based on career plans [22] and residents spending increasing amounts of time on ambulatory rotations[26], more residency programs will likely implement curricula similar to the LSCs in the near future. While there remain some opportunities for growth, the program framework we described here can be useful for the implementation of an ambulatory subspecialty continuity experience for residents.

## Supplementary Material

Supplemental Material
