# Supplementary material for: Evaluation of a longitudinal subspecialty clinic for internal medicine residents
Source: Med Educ Online. 2021 Jul 29;26(1):1955429. doi: 10.1080/10872981.2021.1955429 (PMC8330775; doi:10.1080/10872981.2021.1955429)
Supplement: Supplemental Material [file ZMEO_A_1955429_SM4076.zip › Supplementary/LSC Preceptor Survey_editable.docx]

LSC Preceptor Survey

Start of Block: Introduction

Q1 We are seeking to evaluate the effectiveness of the Longitudinal Subspecialty Clinic (LSC) program and to determine best practices for the LSC program's logistics and curriculum.  We are conducting surveys of UCSF internal medicine residents, alumni, and faculty preceptors who have participated in the LSCs.  The results of the surveys will help us improve the LSC program.

**This is an anonymous survey, and your responses will be kept confidential.**

End of Block: Introduction

Start of Block: Demographics

Q2 What is your faculty rank at UCSF?

- Clinical Instructor
- Assistant Professor
- Associate Professor
- Professor
- Other (please specify) ________________________________________________

Q3 What subspecialty do you work in as an LSC preceptor?

- Allergy and Immunology
- Cardiology
- Endocrinology
- Gastroenterology or Hepatology
- Geriatric Medicine
- Hematology-Oncology
- Hospice and Palliative Medicine
- Infectious Diseases or HIV
- Nephrology
- Pulmonary
- Rheumatology
- Other (please specify) ________________________________________________

Q4 How many years of experience do you have as an LSC preceptor, **including the 2018-2019 year**?

|  | 0 | 5 | 10 | 15 | 20 |
| --- | --- | --- | --- | --- | --- |

| Years | 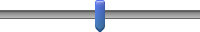 |
| --- | --- |

Q5 How many residents did you precept in an LSC **during the 2018-2019 year**?

|  | 0 | 1 | 2 | 3 | 4 | 5 |
| --- | --- | --- | --- | --- | --- | --- |

| Residents | 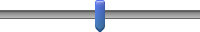 |
| --- | --- |

End of Block: Demographics

Start of Block: Career Choice, Mentorship, and Education

| 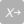 |
| --- |

Q6 From your perspective, how **effective** is the LSC experience in enabling residents to:

|  | N/A | Not at all  (1) | Slightly  (2) | Somewhat  (3) | Very  (4) | Extremely  (5) |
| --- | --- | --- | --- | --- | --- | --- |
| Explore career options in their subspecialty of interest. |  |  |  |  |  |  |
| Gain exposure to ambulatory subspecialty practice. |  |  |  |  |  |  |
| Form a meaningful mentoring relationship with their assigned faculty preceptor. |  |  |  |  |  |  |
| Obtain a letter of recommendation for fellowship applications. |  |  |  |  |  |  |
| Gain medical knowledge in their subspecialty of interest. |  |  |  |  |  |  |
| Prepare for fellowship and/or clinical practice. |  |  |  |  |  |  |
| Build their confidence in managing subspecialty conditions in the outpatient setting. |  |  |  |  |  |  |
| Form longitudinal relationships with patients. |  |  |  |  |  |  |

Q7 Do you feel like you got to know your current resident(s) **well enough** to write them a letter of recommendation?

- Yes
- No
- Maybe

Display This Question:

If Do you feel like you got to know your current resident(s) well enough to write them a letter of r... = No

Or Do you feel like you got to know your current resident(s) well enough to write them a letter of r... = Maybe

Q8 Please explain your reasoning for your answer (No/Maybe) to the above question.

________________________________________________________________

________________________________________________________________

________________________________________________________________

________________________________________________________________

________________________________________________________________

End of Block: Career Choice, Mentorship, and Education

Start of Block: Curriculum

Q9
Regarding LSC curriculum, how **frequently** do the following occur in your clinic's LSC over the course of a year?

|  | Never  (1) | Rarely  (2) | Sometimes  (3) | Usually  (4) | Always  (5) |
| --- | --- | --- | --- | --- | --- |
| Your resident attends pre-/post-clinic conference. |  |  |  |  |  |
| You assign readings to your resident. |  |  |  |  |  |
| Your resident attends didactics (in-person lectures). |  |  |  |  |  |
| You assign online videos/learning modules to your resident. |  |  |  |  |  |

Q10 In your opinion, what is the **ideal** method for delivering curriculum to a resident in an LSC?

________________________________________________________________

________________________________________________________________

________________________________________________________________

________________________________________________________________

________________________________________________________________

End of Block: Curriculum

Start of Block: Logistics

Q11
Regarding clinic workflow, how **frequently** do the following occur in your clinic's LSC over the course of a year?

|  | Never  (1) | Rarely  (2) | Sometimes  (3) | Usually  (4) | Always  (5) |
| --- | --- | --- | --- | --- | --- |
| Your resident sees patients independently before staffing with you. |  |  |  |  |  |
| Your resident shadows you. |  |  |  |  |  |
| Your resident's patients are scheduled specifically for them ahead of time. |  |  |  |  |  |
| Your resident sees patients off your schedule who are chosen for them based on case diversity, complexity, or educational value. |  |  |  |  |  |
| Your resident sees patients off your schedule solely based on clinic logistics and workflow. |  |  |  |  |  |

Q12 In your opinion, what is the **ideal** workflow for a resident to see patients in an LSC?

________________________________________________________________

________________________________________________________________

________________________________________________________________

________________________________________________________________

________________________________________________________________

Q13 On average, how many patients does your resident see each half-day LSC session?

|  | 0 | 1 | 2 | 3 | 4 | 5 | 6 | 7 | 8 | 9 | 10 |
| --- | --- | --- | --- | --- | --- | --- | --- | --- | --- | --- | --- |

| Patients | 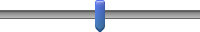 |
| --- | --- |

Q14 In your opinion, what is the **ideal** number of patients for a resident to see each half-day LSC session?

|  | 0 | 1 | 2 | 3 | 4 | 5 | 6 | 7 | 8 | 9 | 10 |
| --- | --- | --- | --- | --- | --- | --- | --- | --- | --- | --- | --- |

| Patients | 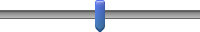 |
| --- | --- |

Q15 On average, how many patients do your residents see **more than once** over the course of a year?

|  | 0 | 5 | 10 | 15 | 20 | 25 | 30 |
| --- | --- | --- | --- | --- | --- | --- | --- |

| Patients | 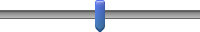 |
| --- | --- |

| 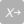 |
| --- |

Q16
From your perspective, how **frequently** do you feel like your resident's LSC experience is interrupted because of competing demands (coverage, elective time, vacation, etc) over the course of a year?

- Never (1)
- Rarely (2)
- Sometimes (3)
- Usually (4)
- Always (5)

Q17 What is the impact of having a longitudinal resident on your efficiency in clinic?

- Significantly Decreases (1)
- Slightly Decreases (2)
- No Change (3)
- Slightly Increases (4)
- Significantly Increases (5)

Q18 Please explain the reason for this impact on your efficiency in clinic:

________________________________________________________________

________________________________________________________________

________________________________________________________________

________________________________________________________________

________________________________________________________________

Q19 Currently, the option to participate in an LSC is offered to residents for only **one** year due to availability constraints in the subspecialty clinics.  In light of this, please answer the following:


I would **prefer** that the option for residents to participate in an LSC occur during:

- R2 year (please explain why) ________________________________________________
- R3 year (please explain why) ________________________________________________

Q20 If having an LSC in the R2 **and** R3 years were possible from a logistical perspective, which of the following would you prefer?

- My resident has an LSC in both years (stays in my LSC for both years)
- My resident has an LSC in both years (switches to a different LSC for the R3 year)
- My resident has an LSC for only one year

End of Block: Logistics

Start of Block: Overall Ratings and Free Response Questions

Q21 Please rate how **satisfied** you are with your overall experience as an LSC preceptor.

- Not at all (1)
- Slightly (2)
- Somewhat (3)
- Very (4)
- Extremely (5)

Q22 Please rate the overall **educational value** of the LSC experience for residents.

- Poor (1)
- Fair (2)
- Good (3)
- Very Good (4)
- Excellent (5)

Q23 What are **strengths** of the LSCs?

________________________________________________________________

________________________________________________________________

________________________________________________________________

________________________________________________________________

________________________________________________________________

Q24 What are **areas for improvement** for the LSCs?

________________________________________________________________

________________________________________________________________

________________________________________________________________

________________________________________________________________

________________________________________________________________

Q25 What else would you like to share with us about the LSC experience?

________________________________________________________________

________________________________________________________________

________________________________________________________________

________________________________________________________________

________________________________________________________________

End of Block: Overall Ratings and Free Response Questions
